# The adverse effect profile of acitretin in a pediatric dermatology population—Longitudinal cohort study and recommendations for monitoring

**DOI:** 10.1016/j.jaad.2020.03.082

**Published:** 2020-12

**Authors:** Anna Cave, Isabella Plumptre, Jemima E. Mellerio, Anna E. Martinez, Veronica A. Kinsler

**Affiliations:** a Great Ormond St Hospital for Children and UCL GOS Institute of Child Health, London, United Kingdom; b Guys and St Thomas' Hospital, London, United Kingdom; c Mosaicism and Precision Medicine Laboratory, The Francis Crick Institute, London, United Kingdom

*To the Editor:* The clinical benefit of acitretin has been amply shown in the treatment of disorders of keratinization in childhood, particularly in psoriasis and ichthyosis. The adverse effects (AEs) of acitretin are well studied in adults, and monitoring guidelines were issued by the British Association of Dermatologists.^1^ However, AEs in childhood are less well studied, particularly in non-psoriasis cohorts.

A retrospective case note review was undertaken of all 174 patients prescribed acitretin between 1993 and 2015. Patient variables collected were diagnosis; demographics; age at starting acitretin; length of time monitored while receiving acitretin (as measured by age at stopping treatment or age of transfer to adult services if still receiving the medication); starting, maximal, and final doses; and AEs. Children were usually seen by a dermatologist on a three-monthly basis and none were lost to follow-up in the study period. Clinical AEs were defined as any reported clinical symptom that had arisen since starting and could be attributed to acitretin. Laboratory AEs were defined as hepatic transaminase levels twice the upper limit of the normal range for age, and/or alkaline phosphatase levels at least 1.2-fold the upper limit of normal range for age, and/or triglyceride levels greater than 2.3 mmol/L. Primary outcome measures (clinical and laboratory AEs) were modeled with respect to 5 patient variables (sex, diagnosis, age at starting, dose/kg at starting, and length of time receiving acitretin) by multiple logistic regression (SPSS, version 22; SPSS Inc, Chicago, IL). A Bonferroni correction for multiple testing was applied, reducing the level of significance to *P* < .005. Response to treatment was not a primary outcome but has been recorded here for comparability with other studies.

Cohort data are shown in Table I. There were no fatal or irreversible AEs documented due to acitretin. Clinical AEs were reported in 24%, leading to permanent cessation of treatment in 10% of the total cohort, although this overlapped with lack of adequate response to the medication—in other words, the balance of beneficial and adverse clinical AEs was important and not easily measurable. Laboratory AEs occurred in 22%, leading to permanent cessation in 4% of the total cohort. Importantly, laboratory AEs were very rare after 2 years of uneventful treatment (Supplemental Figure 1; available via Mendeley at https://data.mendeley.com/datasets/x7cp29vtgk/draft?a=11828aec-97a2-46f7-8dd2-49a213c6ddc0). Reduced bone density was seen in 3 patients with ichthyosis, a known risk factor for vitamin D deficiency, but was not routinely screened for. There were no significant associations between clinical or laboratory AEs and the 5 patient variables. Half of those children who had acitretin stopped for any AE subsequently had the drug restarted.

**Table I:** Cohort demographics and incidence of clinical and laboratory adverse effects potentially due to acitretin therapy

Demographics of the total cohort (N = 174)	Count∗
Male/female, n	90/84
Age at starting acitretin, y, mean ± SEM (range)	8.1 **±** 0.4 (0.0-18.78)
Mean duration of treatment, y, mean ± SEM (range)	3.5 ± 0.3 (0.02-17.58)
Mean starting dose, mg/kg, mean ± SEM (range)	0.42 ± 0.01 (0.15-0.68)
Mean maximum dose, mg/kg, mean ± SEM (range)	0.45 ± 0.02 (0.14-1.24)
Clinical diagnostic groupings, n/total (%)	
Congenital ichthyosis	94/172 (54.7)
Psoriasis	50/172 (29.1)
Other	14/172 (8.1)
Palmoplantar keratoderma	5/172 (2.9)
Eczema	4/172 (2.3)
Immunodeficiency	4/172 (2.3)
Pityriasis rubra pilaris	1/172 (0.6)
Missing data on diagnosis	2/172 (1.2)
Clinical AEs, all reversible, n/total (%)	
Increased skin irritation, fragility, or rash	18/174 (10.3)
Dry lips	16/174 (9.2)
Nausea	3/174 (1.7)
Tiredness/malaise	2/174 (1.1)
Mood swings	1/174 (0.6)
Hair thinning	1/174 (0.6)
Abdominal pain	1/174 (0.6)
Clinical AEs of any type, n/total (%)	42/174 (24.1)
Missing data on clinical AEs	0/174
Laboratory AEs, all reversible, n/total (%)	
Abnormal alkaline phosphatase level alone	10/170 (5.9)
Abnormal alanine transaminase level alone	4/170 (2.4)
Abnormal alanine transaminase and alkaline phosphatase level	1/170 (0.6)
Abnormal triglyceride levels alone	17/170 (10)
Abnormal liver function test results (alanine transaminase or alkaline phosphatase) and triglyceride levels	4/170 (2.4)
Laboratory AE but missing detail on type	2/170 (1.2)
Laboratory AEs of any type	38/170 (22.4)
Missing data on laboratory AEs	4/174
Reasons for stopping acitretin during study period, temporarily or permanently, n/total (%)	
Limited or no clinical improvement	33/172 (19.2)
Clinical AEs (other than worsening of skin disease)	21/172 (12.2)
Sustained clinical improvement	17/172 (9.9)
Worsening of skin disease	10/172 (5.8)
Laboratory AEs	7/172 (4.1)
Other causes	3/172 (1.7)
Total stopped acitretin due to any cause, n/total (%)	91/172 (52.9)
Missing data on stopping	2/174

*AE,* Adverse event; *SEM,* standard error of the mean.

∗ Where the total cohort number does not equal 174, this is due to missing data.

In this cohort, acitretin was therefore a safe drug at the dose used and for the duration of follow-up, subject to clinical and laboratory monitoring. Incidence of AEs was unaffected by sex, diagnosis, age at starting, dose, or duration of therapy; however, laboratory AEs after 2 years of uneventful treatment were uncommon. The overall pattern of AEs is comparable to that in previous studies of acitretin in children,^2^, ^3^, ^4^, ^5^ as is the number of children stopping acitretin due to clinical AEs.^4^ As reported previously, minor changes in blood indices have been associated with acitretin use and do not tend to lead to changes in therapy.^5^

**Fig 1:**
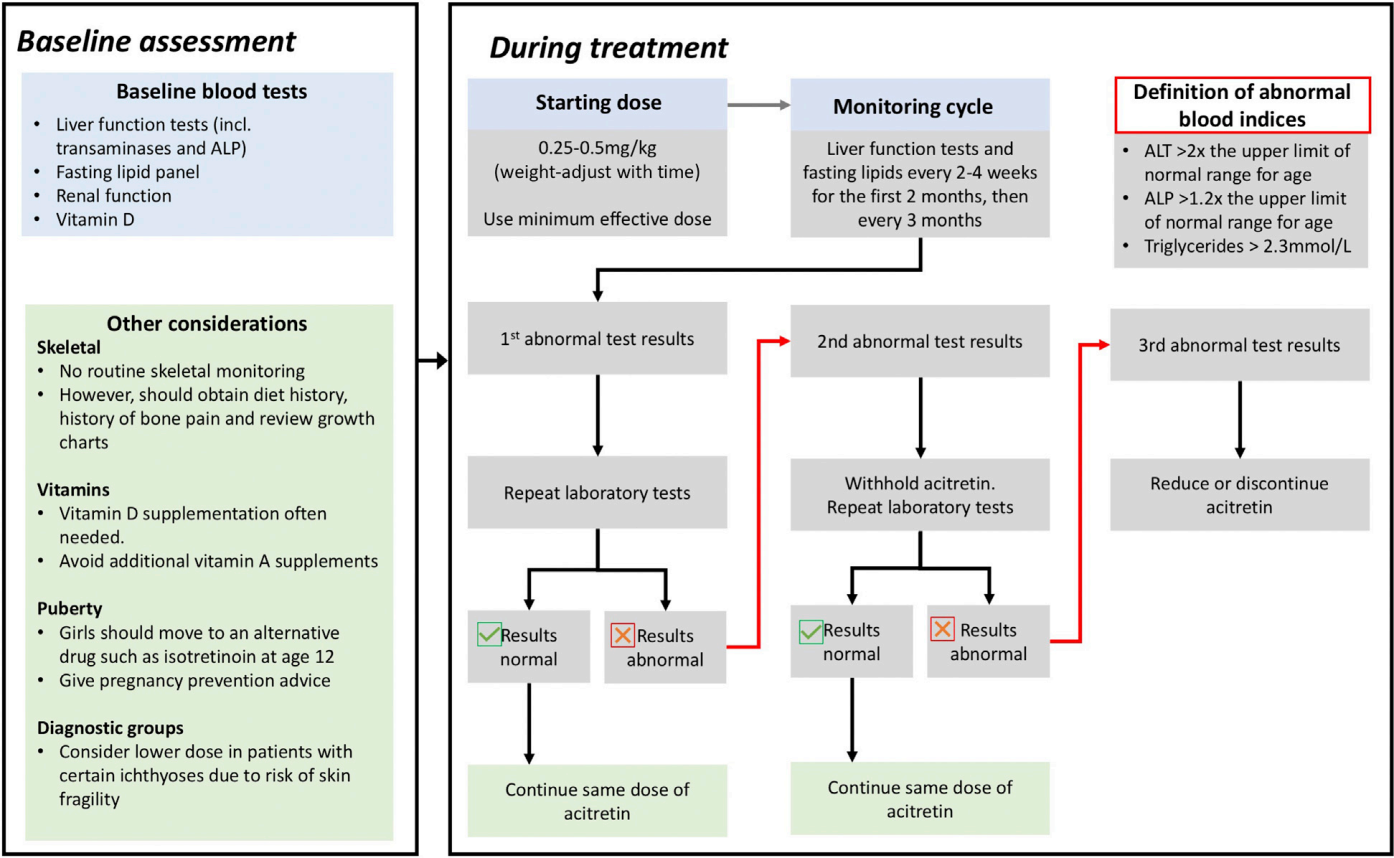
Suggested guidelines for monitoring the use of acitretin in children in association with the published guidelines in adults. *ALP,* Alkaline phosphatase; *ALT,* alanine transaminase.

In conclusion, our current practice is based on the published adult guidelines^1^ and these findings, and is summarized in Fig 1.
